# Knock-Down of Gossypol-Inducing Cytochrome P450 Genes Reduced Deltamethrin Sensitivity in *Spodoptera exigua* (Hübner)

**DOI:** 10.3390/ijms20092248

**Published:** 2019-05-07

**Authors:** Muhammad Hafeez, Sisi Liu, Saad Jan, Le Shi, G. Mandela Fernández-Grandon, Asim Gulzar, Bahar Ali, Muzammal Rehman, Mo Wang

**Affiliations:** 1 Hubei Insect Resources Utilization and Sustainable Pest Management Key Laboratory, College of Plant Science and Technology, Huazhong Agricultural University, Wuhan 430070, Hubei, China; drhafeez@webmail.hzau.edu.cn (M.H.); leshi@webmail.hzau.edu.cn (L.S.); bahardirvi@webmail.hzau.edu.cn (B.A.); 2 Laboratory of medicinal Biophysical Chemistry, College of Science, Huazhong Agricultural University, Wuhan 430070, Hubei, China; 3 Department of Agriculture Entomology Section, Bacha Khan University, Charsadda 24630, Pakistan; drsaadjan@bkuc.edu.cn; 4 Natural Resources Institute, University of Greenwich, Chatham Maritime, Kent, Gillingham ME4 4TB, UK; m.fernandez-grandon@greenwich.ac.uk; 5 Department of Entomology, Pir Mehr Ali Shah Arid Agriculture University, Rawalpindi, Punjab 46000, Pakistan; asim@uaar.edu.pk; 6 MOA Key Laboratory of Crop Ecophysiology and Farming System in the Middle Reaches of the Yangtze River, College of Plant Science and Technology, Huazhong Agricultural University, Wuhan 430070, Hubei, China; muzammal@webmail.hzau.edu.cn

**Keywords:** *Spodoptera exigua*, deltamethrin tolerance, gossypol, detoxification, midgut P450 genes, RNA interference

## Abstract

Plants employ an intricate and dynamic defense system that includes physiological, biochemical, and molecular mechanisms to counteract the effects of herbivorous attacks. In addition to their tolerance to phytotoxins, beet armyworm has quickly developed resistance to deltamethrin; a widely used pyrethroid insecticide in cotton fields. The lethal concentration (LC_50_) required to kill 50% of the population of deltamethrin to gossypol-fed *Spodoptera exigua* larvae was 2.34-fold higher than the control group, suggesting a reduced sensitivity as a consequence of the gossypol diet. Piperonyl butoxide (PBO) treatment was found to synergize with deltamethrin in gossypol-fed *S. exigua* larvae. To counteract these defensive plant secondary metabolites, beet armyworm elevates their production of detoxification enzymes, including cytochrome P450 monooxygenases (P450s). Gossypol-fed beet armyworm larvae showed higher 7-ethoxycoumarin-O-deethylase (ECOD) activities and exhibited enhanced tolerance to deltamethrin after 48 and 72 h when compared to the control. Moreover, gossypol pretreated *S. exigua* larvae showed faster weight gain than the control group after transferring to a deltamethrin-supplemented diet. Meanwhile, gossypol-induced P450s exhibited high divergence in the expression level of two P450 genes: *CYP6AB14* and *CYP9A98* in the midgut and fat bodies contributed to beet armyworm tolerance to deltamethrin. Knocking down of *CYP6AB14* and *CYP9A98*, via double-stranded RNAs (dsRNA) in a controlled diet, rendered the larvae more sensitive to the insecticide. These data demonstrate that generalist insects can exploit secondary metabolites from host plants to enhance their defense systems against other toxic chemicals. Impairing this defense pathway by RNA interference (RNAi) holds a potential to eliminate the pest’s tolerance to insecticides and, therefore, reduce the required dosages of agrochemicals in pest control.

## 1. Introduction

Plants respond to herbivory through an intricate and dynamic defense system that includes physiological, biochemical, and molecular mechanisms to counteract the effects of insect attacks [1,2]. The arms race for the insects to overcome these changing plant defenses leads to implications for their behavior and fitness [3]. Previous studies revealed that different types of plant secondary metabolites have been shown resistance by acting as feeding deterrents, toxins, and growth inhibitors against several insect orders including Coleoptera, Lepidoptera, and Hymenoptera [4,5,6]. Gossypol and related sesquiterpene aldehydes are the main secondary metabolites in cotton plants and have been shown to possess insecticidal activities and fungistatic properties [7,8].

However, the phytophagous insect does not act as a passive victim. It has been documented that, to detoxify or tolerate a variety of toxic compounds produced by plants or other sources, insects have evolved sophisticated defense system to elevate the activities of detoxification enzymes, such as esterase, glutathione S-transferases, and cytochrome P450 monooxygenases [9,10,11]. Cytochrome P450s (CYPs) are a large gene family of multifunctional enzymes which are involved in both metabolic detoxification of plant secondary metabolites and chemical insecticides in herbivorous insects [10,12]. The insect detoxification system mainly consists of transferring, metabolizing, and excreting of toxic compounds [13]. A variety of P450 substrate recognition sites induce the metabolic malleability, meaning that manifold P450 genes can metabolize a single substrate, or one P450 gene can metabolize a number of different substrates [14]. A large number of detoxification-related P450s genes from insects have been isolated and identified, many of which belong to the CYP6 family [14,15]. For example, gossypol-induced P450s showed high divergence, of which at least five of them (*CYP6AE11*, *CYP321A1*, *CYP9A12*, *CYP9A14*, and *CYP6B7*) contributed to the tolerance of deltamethrin insecticide in cotton bollworm (*Helicoverpa armigera*) [16]. Additionally, *CYP6AS* in the honey bee, *Apis mellifera*, could metabolize the flavonoid quercetin [17]. Further studies have shown that the expression of five P450 genes (*CYP9A14*, *CYP337B1*, *CYP9A12*, *CYP6AE11*, and *CYP6B7*) in the resistant strains of *H. armigera* was significantly higher in response to fenvalerate pesticides [18,19]. Cross-resistance of alpha-cypermethrin after ingestion of xanthotoxin in *Helicoverpa zea* and high expression response of P450s genes (*CYP6B2*, *CYP6B6*, *CYP6B7*, *CYP6B8* and *CYP321A1*) were observed in quercetin fed larvae of *H. armigera* [11,20] In addition, insecticide resistance was found to be different in populations fed on different host plant species [21], indicating that the host plant and its secondary metabolites may affect insect susceptibility to pesticides. As the main phytotoxin of cotton, gossypol plays an important role in shaping the defensive state of beet armyworm.

For decades chemical pesticides have been a common approach for controlling vector-borne diseases and insect pests in crop systems. Synthetic pyrethroids are considered to be effective insecticides for insect pest control and play a significant role in reducing crop losses caused by herbivores insects [22]. However, extensive usage of insecticides has lowered their efficacy due to the development of insect resistance. Increasingly, concern is also raised about the environmental pollution and human health issues that they cause. Beet armyworm, *Spodoptera exigua*, is a major insect pest that feeds on many economically important crops globally. In China, it is particularly responsible for extensive losses in the vegetable, ornamental plant, and cotton sector [23]. Chemical insecticides have been extensively used for control of this pest which has led to strong selection of strains resistant to pyrethroids and more recently developed chemistries [24,25,26,27]. This, in turn, has led to the failure of traditional control strategies in the field [28]. A newer and promising strategy that could have a significant impact on the economy of agriculture is the implementation of RNA interference (RNAi). RNAi is an efficient technique in the study of insect resistance and could play a significant role in pests control [24]. In numerous insects, RNAi techniques have been applied to partially silence P450 genes responsible for insecticide resistance, such as *CYP9A14* [16], *CYP6AE14* [12] or *CYP9A21v3* [24]. In addition, transgenic plants expressing double-stranded RNAs (dsRNA) have exhibited effect control for pests. The expression of P450 genes has been found to decrease dramatically when the larvae of *H. armigera* were fed on transgenic cotton plants expressing *CYP6AE14* as a result of physiological impact, such as loss of appetite, growth retardation, and ultimately death [12]. RNAi has also been used to study the *Escherichia coli*-expressed dsRNA of *SeCHSA* in *S. exigua*. When *S. exigua* larvae were fed *E. coli* expressing the dsRNA of *SsCHSA* (chitin synthase gene A), it led to significant reductions in survival rates of the *S. exigua* [29]. These advances in our understanding of RNA interference as a tool in insect pest regulation may pave the way for its development for wider, commercial usage.

This study was conducted to explore the effect of plants secondary metabolites on tolerance of *S. exigua* larvae to deltamethrin. We measure the P450 midgut detoxification enzyme activity toward specific substrates to demonstrate the biochemical characteristics of gossypol-stimulated tolerance to deltamethrin. In addition, we examined the potential role of cytochrome P450 genes in detoxification of plant secondary metabolites and pyrethroid insecticides in *S. exigua* larvae. The tissue-specific expression patterns of CYP450 genes and its potential role in synthetic pyrethroid insecticide detoxification were studied by quantitative real-time-PCR. RNA interference was used to downregulate the *CYP6AB14* and *CYP9A98* genes. These results provide important insight into the role of CYP450 genes in detoxification of gossypol-pretreated deltamethrin resistance and may be essential in the development of novel methods for the control of *S. exigua*.

## 2. Results

### 2.1. Induced Effect of Gossypol to Deltamethrin Tolerance and Synergism Assessment

To find out the effect of plant secondary metabolites on insecticide tolerance, we examined the induced effect of gossypol-supplemented diet on the sensitivity of *S. exigua* larvae to deltamethrin, a widely used pyrethroid insecticide in many crops including cotton. We found that deltamethrin showed lowered toxicity to the *S. exigua* larvae fed on an artificial diet supplemented with 1% gossypol per gram of diet than it did in the gossypol-free control group. We also found that lethal concentration to 50% (LC_50_) value of deltamethrin to the gossypol-fed third instar *S. exigua* larvae was higher (1.704 mg/L) while the lower LC_50_ value was observed for the control diet (0.887 mg/L) (Table 1). Furthermore, the synergism assay indicated that PBO treatment as a synergist effectively increased the deltamethrin efficacy in gossypol-fed larvae with a synergism ratio 1.7 as compared to the control group in which PBO showed low synergism to deltamethrin. These results showed that a gossypol-supplemented diet increased the level of tolerance to deltamethrin insecticide.

### 2.2. Effect of Gossypol Diet on Larval Body Weight

**Figure 1:**
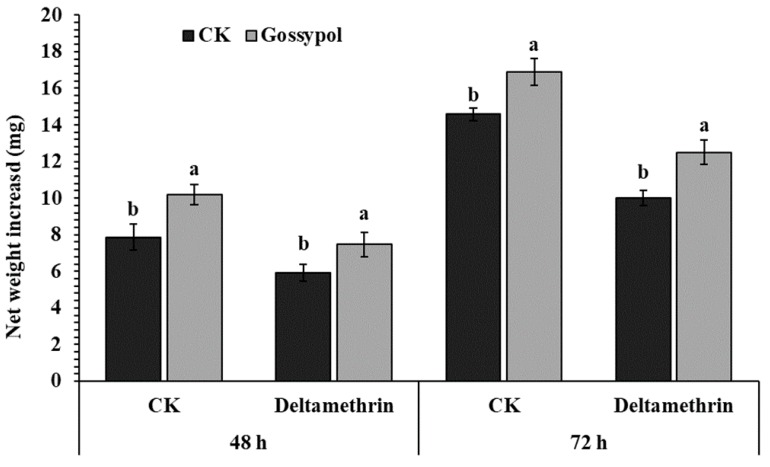
Net weight increase in gossypol-pretreated larvae on deltamethrin-supplemented diet. The early third instar larvae had previously fed on control (CK) or 1 mg/g gossypol-supplemented (Gossypol) diet for 1 day; after recording the initial weight, two independent groups of each treatment were transferred to 0.887/L deltamethrin-supplemented diet and control diet, respectively, weight increases were recorded 2 days later. Error bars represent standard deviation. Different letters above bars indicate significant differences (*p* < 0.05) according to the Student’s *t*-test.

To observe the induced effect of gossypol on the net larvae weight of *S. exigua*, gossypol-supplement and control diet was fed to third instar larvae for one day before being transferred to a diet containing LC_50_ concentration of deltamethrin. Net weight increased in gossypol-pretreated larvae on deltamethrin-supplemented diet significantly faster than was observed in the control diet after 48 h, while the most obvious different body weight gain increased in gossypol-pretreated larvae on deltamethrin-supplemented diet and control treatment at 72 h (Figure 1).

### 2.3. Effect of Gossypol on Midgut P450 Activity

**Figure 2:**
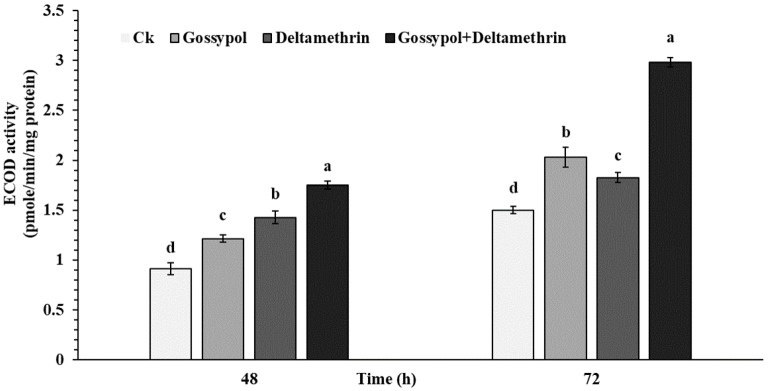
Induced effects of gossypol on beet armyworm tolerance to deltamethrin and O-deethylase activity of P450s after 48 and 72 h in forth instar. The early forth instar larvae were transferred into new sterilized plastic cups containing artificial diets supplemented with 0.1 % gossypol, LC_50_ concentration of deltamethrin 0.887mg/L for 48 and 72 h or 0.1 % gossypol for 24 h followed by deltamethrin for 48 and 72 h. Data shown are means ± SE derived from three biological replicates. Different letters above bars indicate significant differences (*p* < 0.05) according to the Tukey HSD test.

To determine the biochemical properties of the gossypol-induced effect on deltamethrin tolerance to the fourth instar *S. exigua* larvae, the activity of P450 detoxification enzyme in midguts was analyzed (Figure 2). Results indicate that P450 detoxification enzyme activity in the midgut of *S. exigua* larvae was significantly elevated in artificial diets supplemented with 0.1 % gossypol, LC_50_ concentration of deltamethrin 0.887mg/L for 48 and 72 h or 0.1 % gossypol for 24 h followed by deltamethrin for 48 and 72 h when compared to the control group (Figure 2). In addition, significantly higher activity was observed in 0.1 % gossypol for 24 h followed by deltamethrin for 48 and 72 h when compared to other treatments, respectively.

### 2.4. Effect of Gossypol, Flavone, and Deltamethrin on Expression Response of P450 Genes

**Figure 3:**
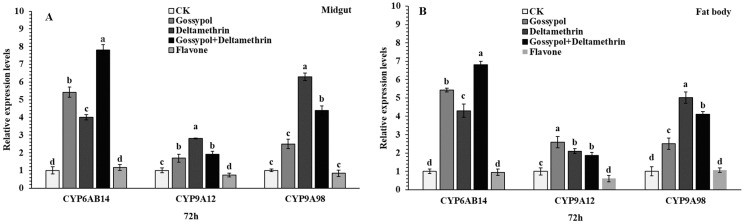
Effect of gossypol on beet armyworm tolerance to deltamethrin and relative expression levels of three P450s genes in midgut (**A**) and fat body (**B**) of *S. exigua*. Late third instar larvae were transferred into new sterilized plastic cups containing artificial diets supplemented with 0.1 % gossypol, LC_50_ concentration of deltamethrin 0.887mg/L for 72 h or 0.1 % gossypol for 24 h followed by deltamethrin for 72 h. Data shown are means ± SE derived from three biological replicates. The transcription levels of three P450s genes determined by quantitative real-time PCR, normalized to three reference genes Different letters above bars indicate significant differences (*p* < 0.05) according to the Tukey HSD test.

The induced effect of gossypol to deltamethrin tolerance on the expression level of *CYP6AB14*, *CYP9A12*, and *CYP9A98* was examined in midguts and fat bodies of *S. exigua* (Figure 3). Fourth instar *S. exigua* larvae were exposed to plant allelochemicals 1.0 % gossypol, 1.0 % flavone, and deltamethrin insecticide for 72 h and gossypol for one day followed by deltamethrin for 72 h in two tissues (midguts and fat bodies) of *S. exigua* larvae. The expression level of *CYP6AB14* was significantly increased in the midgut (gossypol: 5.4, deltamethrin: 4.0 and gossypol+deltamethrin: 7.8-fold) and fat bodies (gossypol: 5.4, deltamethrin: 4.3 and gossypol+deltamethrin: 6.8-fold). In addition, a 7.8-fold higher *CYP6AB14* expression level was observed for gossypol+deltamethrin treated fourth instar larvae in midguts after 72 h compared with the control group (Figure 3). Similarly, significantly increased *CYP9A12* expression level was also observed in midgut and fat bodies in treated groups as compared with the control groups. Following treatment, steady-state expression level was observed for *CYP9A98* in the midgut (gossypol: 2.49, deltamethrin: 6.29 and gossypol+deltamethrin: 4.39 fold) and fat bodies (gossypol: 2.5, deltamethrin: 5.0 and gossypol+deltamethrin: 4.1 fold) (Figure 3). The maximum increase in *CYP6AB14* expression was observed for gossypol+deltamethrin in midguts and fat bodies. In comparison with marked induction by gossypol, *CYP6AB14*, *CYP9A12*, and *CYP9A98* were weakly induced by flavone, which exerted little or no effect.

### 2.5. Effect of dsCYP6AB14 and dsCYP9A98 on Larval Mortality

**Figure 4:**
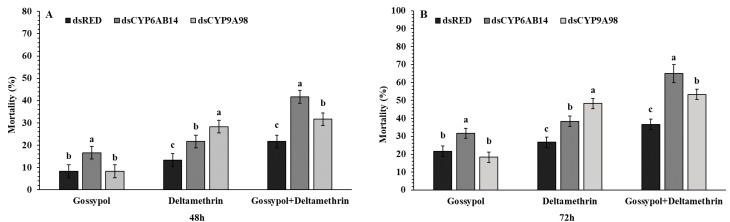
Single effects of *dsCYPAB14* and *dsCYPA98* feeding on the susceptible of fourth instar *S. exigua* larvae. Following the droplet-feeding with *dsCYPAB14+dsCYPA98* or *dsRED* for 24 h the exposed larvae were transferred individually into 12-oriface tissue culture plate containing artificial diets supplemented with 0.1 % gossypol, LC_50_ concentration of deltamethrin 0.887mg/L for 48 (**A**) and 72 h (**B**) or 0.1 % gossypol for 24 h followed by deltamethrin for 48 (**A**) and 72 h (**B**). Data shown are means ± SE derived from three biological replicates. Different letters above bars indicate significant differences (*p* < 0.05) according to the Tukey HSD test.

Mortality of fourth instar larvae of *S. exigua* via droplet-fed *dsRED*, *dsCYP6AB14*, and *dsCYP9A98* for 24 h and followed by feeding on 0.1 % gossypol, LC_50_ concentration of deltamethrin 0.887mg/L for 72 h and then 0.1 % gossypol for 24 h before being transferred to deltamethrin for a further 72 h. In the control group, a standard diet was provided. This result indicated the feeding of *dsCYP6AB14* and *dsCYP9A98 larvae* significantly increased the toxicity of gossypol and deltamethrin. Larval droplet feeding with *dsCYP6AB14* significantly increased the larval mortality caused by gossypol (16.66%), deltamethrin (20%), and gossypol+deltamethrin (41.33%). Similarly, *dsCYP9A98* feeding led to increased mortality in gossypol (8.33%), deltamethrin (28.33%), and gossypol+deltamethrin (31.66%) compared with control *dsRED*, which was for gossypol (8.33%) deltamethrin (13.3%), and gossypol+deltamethrin (21.66%) at 48 h (Figure 4). Increased mortality was also observed at 72 h for *dsCYP6AB14* fed larvae transferred on diet supplemented with gossypol (31.66%), deltamethrin (38.33%), and gossypol+deltamethrin (65%) and for *dsCYP9A98* fed larvae on diet supplemented with gossypol (18.33%), deltamethrin (48.24%), and gossypol+deltamethrin (53.33%) as compared with the *dsRED* control group at 72 h, which showed lower mortality rates for gossypol (21.33%), deltamethrin (26.66%), and gossypol+deltamethrin (36.66%) (Figure 4). The progression of mortality in dsRNA treated normal larvae and mortality assays the mock controls have been shown in Figure S1.

### 2.6. The Combined Effect of Target dsCYP6AB14+dsCYP9A98 Genes on Larval Mortality

**Figure 5:**
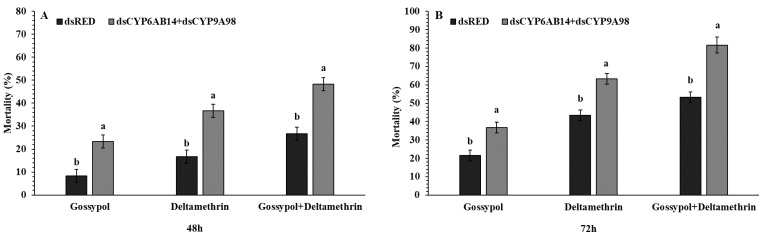
Combined effect of *dsCYPAB14+dsCYPA98* feeding on the mortality of fourth instar *S. exigua* larvae. Following the droplet-feeding with *dsCYPAB14+dsCYPA98* or *dsRED* for 24 h the exposed larvae were transferred individually into 12-oriface tissue culture plate containing artificial diets supplemented with 0.1 % gossypol, LC_50_ concentration of deltamethrin 0.887mg/L for 48 (**A**) and 72 h (**B**) or 0.1 % gossypol for 24 h followed by deltamethrin for 48 (**A**) and 72 h (**B**). Data shown are means ± SE derived from three biological replicates. Different letters above bars indicate significant differences (*p* < 0.05) according to the Student’s *t*-test.

Target genes *dsCYP6AB14+dsCYP9A98* combination was prepared at the final concentration of 1000 ng/µl. After combined application of dsRNA, the larval mortality was significantly increased which showed 23.33%, 36.66%, and 48.33% for gossypol, deltamethrin, and gossypol+deltamethrin, respectively at 48 h compared with the ds-RED which showed 8.33%, 16.66%, and 26.66% for the same treatments (Figure 5). Similarly, mortality of *S. exigua* larvae fed on *dsCYP6AB14+dsCYP9A98* following the exposure to gossypol, deltamethrin, and gossypol+deltamethrin was significantly increased after 72 h showing the mortality rate for gossypol, deltamethrin, and gossypol+deltamethrin of 36.33%, 63.33%, and 81.66%, respectively, compared with the ds-RED as a control treatment (Figure 5). In the end, the data showed a fascinating combined effect of *dsCYP6AB14+dsCYP9A98* on the mortality of *S. exigua* larvae.

### 2.7. Effect of Silencing by dsRNA

**Figure 6:**
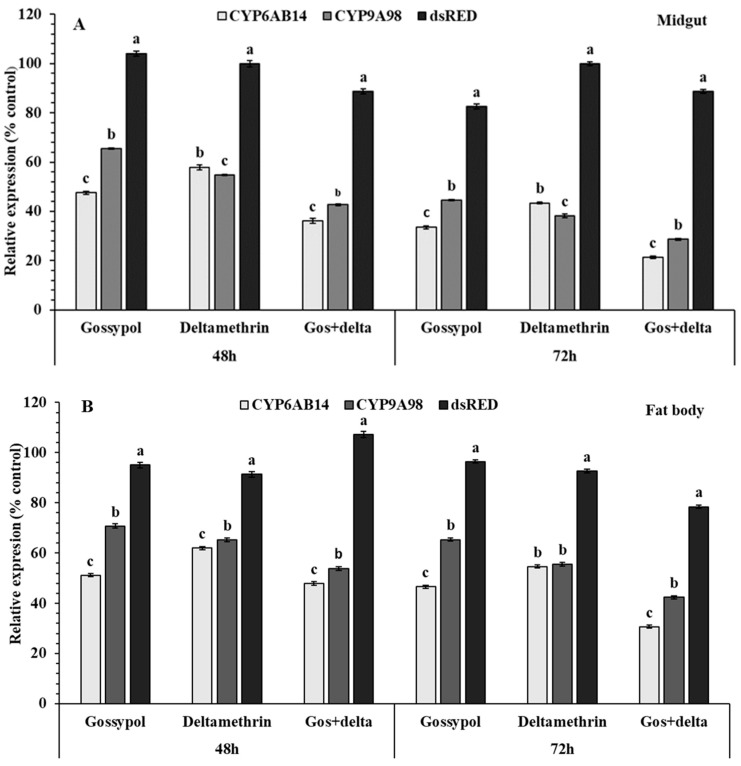
Effect of *dsCYPAB14* and *dsCYPA98* by droplet feeding on relative transcript levels in midguts (**A**) and fat bodies (**B**) after 48 and 72 h (**A**) on the fourth instar *S. exigua* larvae. Following the droplet-feeding with *dsCYPAB14* and *dsCYPA98* or *dsRED* served as a control for 24 h then the exposed larvae were transferred individually into 12-oriface tissue culture plate containing artificial diets supplemented with 0.1 % gossypol, LC_50_ concentration of deltamethrin 0.887mg/L for 48 and 72 h or 0.1 % gossypol for 24 h followed by deltamethrin for 48 and 72 h. Data shown are means ± SE derived from three biological replicates. Different letters above bars indicate significant differences (*p* < 0.05) according to the Tukey HSD test.

To determine whether reduced expression of *dsCYP6AB14* and *dsCYP9A98* have an effect on the susceptibility of *S. exigua* larvae to gossypol and deltamethrin, RNAi-mediated knockdown effect of these genes was performed on fourth instar larvae of *S. exigua* exposed to gossypol and deltamethrin. Twenty-four-hour post-treatment to dsRNA via droplet feeding following exposure on gossypol, deltamethrin, or gossypol+deltamethrin led to significant reductions in the expression levels of *dsCYP6AB14* and *dsCYP9A98* in the midgut and fat bodies at 48 and 72 h, respectively (Figure 6). Furthermore, QRT-PCR results showed that droplet feeding of the combined target genes (*dsCYP6AB14+dsCYP9A98*) significantly reduced the relative expression levels than the ds-RED control or the individual dsRNA treatments in the midguts and fat bodies after 48 and 72 h (Figure 6). This result validated that RNAi effectively suppressed the expression of *CYP9A105* in *S. exigua* exposed larvae under the conditions employed.

## 3. Discussion

The plant biochemical pathway leads to the production of a range of toxic compounds in response to the attack from pathogens or phytophagous insects [30,31]. Variation in defensive constituents of plant response to insect attack presents a variable challenge for insect herbivores, which in turn, affects their fitness and behavior [32,33]. Phytophagous insects protect themselves against these phytotoxic compounds by inducing detoxification genes in response to host plant secondary metabolites for their optimal growth and survival [14]. Usually, a P450 group may metabolize a single substrate in an alternate position or occasionally multiple substrates may be metabolized by a single P450 [14,34].

By ingesting one or more toxic compounds from host plants, phytophagous insects gain an enhanced tolerance to other toxic plant secondary metabolites and chemicals with directly pesticidal properties. The ability to gain tolerance based on post-exposure allows the insects to be highly adaptable to changes in their environment to acquire food from potentially harmful sources. Here we examined the effect of diet incorporation of plant allelochemicals on tolerance of *S. exigua* larvae to frequently used insecticides, deltamethrin in the field. In the present study, larvae of *S. exigua* that ingested gossypol, significantly enhanced tolerance to deltamethrin insecticide (Table 1). Present results support previous studies regarding gossypol pretreatment enhancing deltamethrin tolerance in lepidopteran, *Helicoverpa armigera* [16]. *H. armigera* has also been shown to display increased tolerance to lambda-cyhalothrin following exposure to quercetin in the larval diet [11]. In a similar approach with *Helicoverpa Zea* using a xanthotoxin pretreatment, it was shown that larvae demonstrated greater tolerance to alpha-cypermethrin [35] and four plant allelochemicals flavone, coumarin, DIMBOA (2,4-Dihydroxy-7-methoxy-1,4-benzoxazine-3-one), and visnagin significantly reduced larval sensitivity to methomyl in the larvae of *Helicoverpa armigera* [36]. In addition to Lepidoptera species, a similar phenomenon has also been observed in bee species (Hymenoptera: Apidae) using quercetin pretreatment to decrease sensitivity to tau-fluvalinate [37]. This is conceivable because of in response to selective agrochemicals; some detoxification enzymes, particularly P450s, have evolved the capability to metabolize synthetic insecticide in addition to their specific activity towards plant secondary metabolites.

It has been demonstrated that enhanced activity of detoxification P450 enzymes represents an important biochemical mechanism which metabolizes a wide array of toxic compounds including plant secondary metabolites and pesticides in phytophagous insects [14]. For example, numerous studies have revealed that the midguts and fat bodies of insect larvae play a dominant role in xenobiotic metabolism, and, therefore, detoxification activities of P450 enzymes may be highly expressed in these organs [14,38]. In this study, our results indicated that higher activity of the P450 detoxification enzyme was found in *S. exigua* larvae exposed to gossypol+deltamethrin after 48 and 72 h (Table 2). The results of the current study support previous work on various phytophagous insects, e.g., *Spodoptera liture* [16], *H. armigera* [11], who found that the acquired resistance of the plant defense chemicals is positively correlated with the resistance to some insecticides. In addition, exposure to PBO, an important P450 synergist can inhibit the activity of P450 enzymes in phytophagous insects [39,40,41]. In this investigation, PBO showed synergetic effects on the toxicity of deltamethrin to the gossypol-fed larvae of *S. exigua*, thus, the results of the P450 synergist suggested that resistance to gossypol and deltamethrin is P450-mediated. Building on this evidence, these data suggest that the increased tolerance in *S. exigua* may result from the ability of these plant allelochemicals to induce detoxification enzymes, mainly P450s, which may contribute to the adaptation of polyphagous herbivores to the diverse allelochemicals encountered from their broad range of host plants.

Building on this evidence, these data support the case that the two gossypol-induced P450 genes are demonstrations of the evolutionary relationship between plants allelochemicals and pesticide detoxification.

In the present study, two cytochrome P450 genes induced in *S. exigua* larvae fed on gossypol-supplemented diet were shown to help metabolize phytotoxins and increase resistance to insecticides. Of specific importance, QRT-PCR results demonstrated that these two of gossypol-induced P450 genes (*CYPAB14* and *CYP9A98*) were related to deltamethrin resistance (Figure 1). This builds on and takes forward those findings by demonstrating the effect of host plant secondary metabolites to induce detoxification enzymes that lead to enhanced insecticide tolerance in *S. exigua*. Detail of similar findings has been documented in previous studies. Gossypol-induced P450 genes (*CYP6AE11*, *CYP321A1*, *CYP9A12*, *CYP9A14*, and *CYP6B7*) have been documented by previous studies for cotton bollworm increased tolerance to deltamethrin [16]. Cytochrome P450 gene (*CYP6AE14*) was highly expressed in the midgut of cotton bollworm larvae exposed to a gossypol-supplemented diet [42]. Three P450 genes (*CYP6B6*, *CYP6B8*, and *CYP321A1*) have also shown up-regulation in quercetin-fed larvae of *H. armigera* which reduced the sensitivity to lambda-cyhalothrin [11]. Previous studies established that *CYP9A105*, *CYP9A40*, and *CYP6AB14* are induced by deltamethrin, methoxyfenozide, and plant allelochemicals in *S. litura* and *S. exigua* [41,43,44]. Thus, the presence of highly active defensive compounds in host plant provides a selective pressure for the herbivorous insects to develop a rich pool of defense genes, which is at least one of the reasons why the larvae of beet armyworm have quickly acquired pyrethroid resistance.

RNA interference is a gene-blocking technique which has become well established in the past two decades and has been successfully used in the study of resistance mechanism and P450s function in many insect pests for resistance management [16,24,45,46,47]. RNAi-mediated silencing of insect target genes can be accomplished by introducing dsRNA molecules into the insect to reduce the expression of target genes at a transcriptional level. RNA-mediated interference has widely been used in insects can be achieved through the injection, oral ingestion, or droplet-feeding of dsRNA [16,24,41,48]. In present results, the transcript levels of target genes were significantly decreased when larvae were fed on dsRNA following the exposure to gossypol, deltamethrin, or gossypol+deltamethrin after 48 and 72 h. Our present speculation is consistent with previous works [16,24,41]. Our results showed that RNAi-mediated silencing of *CYP6AB14* and *CYP9A98* significantly increased the mortality of *S. exigua* exposed to gossypol and deltamethrin-supplemented diet individually and gossypol+deltamethrin containing diet as compared with control treatment after 48 and 72h (Figure 4 and Figure 5). These findings of increased mortality are consistent with previous studies on cytochrome genes silencing [24,41,42,49]. Through dsRNA droplet feeding, we were able to lower expression levels of the target genes, which in turn reduced the gossypol-induced resistance to deltamethrin in the population of *S. exigua.*

In conclusion, at present, the rapid development of insecticide resistance of many insects and mites poses a major threat to agriculture all over the world. Based on the results in this study, the exposure of *S. exigua* larvae to gossypol reduces the sensitivity of the pest to a pyrethroid insecticide. We conclude from this that levels of cytochrome P450 detoxification enzymes play a crucial role in *S. exigua* larvae for the adaptation to plant secondary metabolites and synthetic insecticides. Feeding of *dsCYP6AB14* and *dsCYP9A98* increased the larval mortality of *S. exigua* when exposed to a diet containing gossypol and deltamethrin, which indicates that the genes involved in the detoxification of plant secondary metabolites and insecticides can serve as targets for insect pest control. Thus, besides directly impairing the insect growth and development, RNAi technology holds promise for overcoming insecticide resistance in pest populations and reducing the dosage of pesticides needed for effective pest control in the field.

## 4. Materials and Methods

### 4.1. Insect Culture

Beet armyworm (*Spodoptera exigua*) were collected from Jingzhou, Hubei, China in 2003. A colony was established in the laboratory and maintained at 25 °C with 70% relative humidity under a 16 h light:8 h dark photoperiod. Larvae were fed on artificial diet, as previously described by Mao et al. (2007) [42]. The insects were never exposed to any insecticides during the rearing.

### 4.2. Chemicals

Gossypol, 7.ethoxycoumarin, nicotinamide adenine dinucleotide phosphate (NADPH), piperonyl butoxide (PBO), 7-ethoxycoumarin, 7-hydroxycoumarin, Ethylenediaminetetraacetic acid (EDTA) and phenylmethylsulfonyl fluoride (PMSF) were bought from Sigma-Aldrich (St. Louis, MO, USA). Deltamethrin 25% EC (Chimerical formulation) was obtained from Bayer crop science (Hang Zhou, China). Triton X-100 was from Amresco. Dithiothreitol (DTT), glycerol, and Tris were bought from Beijing Solarbio Scientific and Technology Company Beijing, China. Bovine serum albumin was purchased from Beyotime Biotechnology, Jiangsu, China. All chemicals and solvents used were reagent grade.

### 4.3. Preparation of Treatment Diets

The treatment diet was prepared according to the method described by [16] with slight modification; the 0.1% gossypol and 0.1% flavone to be tested were first dissolved in 1% dimethyl sulfoxide (DMSO). The control diet was prepared by adding the same volume of DMSO to the standard artificial diet. To achieve the desired concentration, serial dilutions were prepared from the stock solution of deltamethrin into distilled water. Five serial dilutions for treatment and control groups separately were prepared from the stock solution mixed in distilled water containing 0.1% Triton X-100 and added into the artificial diet before solidification of agar (40–45 °C), mixed gently and poured into new plastic cups (3 cm diameter, 3.5 cm height). The control diet was prepared by adding the same volume of DMSO to artificial diet but without gossypol, flavone or deltamethrin.

### 4.4. Toxicological Analysis of Deltamethrin Tolerance in Larvae

Effects of gossypol uptake on *S. exigua* larvae tolerance to deltamethrin were tested by feeding early third instar larvae on artificial diet containing 0.1% gossypol for 24 before the bioassay. For the control group, the artificial diet was prepared with the same method but without gossypol and flavone. A diet incorporation bioassay was used to determine the toxicity of deltamethrin to third instar larva of *S. exigua* following an established methodology [16]. A stock solution of deltamethrin 25 EC insecticide was prepared in distilled water containing 0.1% Triton X-100 and kept in contact with artificial food. Five serial dilutions (10, 5, 2.5, 1.25, 0.625, and 0.313 mg of deltamethrin/L) for the treatment group and (6, 3, 1.5, 0.75, 0.375, and 0.1875 mg of deltamethrin/L) for control group were then prepared from the stock solution and added into the artificial diet before solidification of agar (40–45 °C). Each was mixed gently and poured into a small, sterilized transparent plastic cup (3 cm diameter, 3.5 cm height). The gossypol-pretreated third-stage larvae and those without pretreatment were transferred onto their diet medium. Each concentration was tested against 60 larvae (three groups of 20 larvae). Mortality was assayed after 24 h of deltamethrin application. The dead larvae were judged using a camel hairbrush, the Larvae were considered dead if they did not respond to the touch of a camel hair brush. The mortality rate is noted as the percentage found dead in each group.

### 4.5. Effect of PBO on the Toxicity of Insecticides

To evaluate if the biochemical basis for tolerance involved P450s, the larvae exposed to the test chemicals were subjected to studies with known pesticide synergist, piperonyl butoxide (PBO). Potential synergy associated with PBO was determined using the bioassay methodology used for deltamethrin toxicity tests with the addition of PBO directly onto the larvae. A solution of PBO was prepared in 1% (*v*/*v*) acetone to the concentration of 25 mg/mL. After *S. exigua* larvae fed on the gossypol-supplemented diet for 24 h, 10 μg of PBO solution was topically delivered onto the dorsal prothorax of each individual larvae using Micro4^TM^ MicroSyringe Pump Controller (USA). After 2 h, the PBO-treated larvae were transferred into new cups containing the artificial diet supplemented with different concentration of deltamethrin (6, 3, 1.5, 0.75, 0.375, and 0.1875 mg of deltamethrin/L). The control group larvae were prepared using the same methodology (including gossypol pretreatment) but without exposure to PBO. Each concentration contained 60 larvae (20 larvae were tested in each of three replicates). Mortality was recorded after 24 h, and the LC_50_ values were calculated [11]. The synergism ratio (SR) was calculated by dividing the LC_50_ of insecticide alone by LC_50_ of insecticide plus synergist [50].

### 4.6. The Effect of 0.1% Gossypol Diet on Bodyweight

To evaluate the effect of gossypol on the growth of *S. exigua*, 120 third instar larvae with uniform size were starved for 2 h and transferred to the sterilized transparent plastic cup (3 cm diameter, 3.5 cm height) containing the artificial diet supplemented with 0.1% gossypol and control (CK) diet without gossypol for 1 day. After 24 h, they were weighed and transferred to a diet containing 0.887 mg⁄ L deltamethrin (a sublethal concentration) for another 24 h. After 2 days of exposure, the net weight increase was recorded.

### 4.7. Samples Preparation for P450 Enzyme Activity

The detoxification enzyme P450 activity in the early fourth instar of *S. exigua* larvae midgut homogenates was assayed. Measurements were taken after they were reared on a diet containing 1.0 mg/g gossypol or no gossypol (control) for one day. After 24 h the exposed larvae were placed into new sterilized plastic cups containing artificial diets supplemented with 0.1 % gossypol, LC_50_ concentration of deltamethrin 0.887mg/L for 48 and 72 h, or 0.1 % gossypol for 24 h followed by deltamethrin for 48 and 72 h. The midgut was removed after 48 or 72 h for further analysis. The midguts from all treatments were extracted by dissection on ice. The dissected midguts were gently shaken to free its contents and washed in a cold aqueous solution containing 1.15% (*w*/*v*) potassium chloride. The crude homogenates of treated and control *S. exigua* midguts were prepared as previously described by Liu et al. [51] with some modification for enzymes activity assay. All experiments were performed in triplicate.

### 4.8. Measurement of P450 Enzyme Activity

The 7-ethoxycoumarin-O-deethylase (ECOD) activity of cytochrome P450 enzyme in the midguts of *S. exigua* larvae using 7-ethoxycoumarin (7-EC) was measured as the substrate according to the method described by (Chen et al. 2017) [11]. Approximately, fifteen midguts third-instar larvae of *S. exigua* were homogenized on ice with 2 mL of homogenization buffer 0.1 M PBS at pH 7.5 containing 1.0 mM EDTA, 1.0 mM phenylmethylsulfonyl fluoride (PMSF), 1.0 mM PTU, 0.1 mM DTT, and 15% glycerol, followed by the centrifugation at 12,000 g for 12 min at 4 °C. The supernatant from centrifuged 2-mL tubes was collected and further used for P450s activity assay. The reaction solutions containing a total of 20 μL of NADPH (10 mM stock solution) and 25 μL of 7-EC (10 mM stock solution) in 685 μL solution of 0.1 M Tris–HCl buffer (pH 7.8) and 250 μL of the enzyme homogenate was added to start the enzyme reaction. The incubation was conducted on a shaker for 15 min at 30 °C, and a 300-μL solution of 15% (w/v) trichloroacetic acid (TCA) was added to terminate the reactions. The mixture in 2-mL tubes was centrifuged at 10,800 g at 4 °C for 2 min, with around 800 μL of supernatant from tubes being transferred to new 2-mL tubes, and a 450-μL solution containing 1.6 M Gly-NaOH buffer (pH 10.5) was added to adjust the pH 10 of resulting extract. The content of 7-hydroxycoumarin in the reaction mixture was measured immediately by using a SPECTRA max GEMINI XS spectrofluorometer (Molecular Devices, USA) with adjusting 356 nm excitation and 465 nm emission filters. A series of different concentrations of 7-hydroxycoumarin were prepared, and standard substance fluorescence values were measured to draw the standard curves. All biochemical assays were conducted at least three replicates with different preparations of enzymes. Each of three replicate consisted of five midguts. Protein concentration was determined using the method described by Bradford [52] but with bovine serum albumin as the standard protein.

### 4.9. Sample Preparation

To determine tissue-specific expression patterns for the target genes, the third instar larvae were transferred into new sterilized plastic cups containing artificial diets supplemented with 0.1 % gossypol, LC_50_ concentration of deltamethrin 0.887mg/L for 72 h or 0.1 % gossypol for 24 h followed by deltamethrin for 72 h and 1.0 mg/g DMSO for the control group. After 72 h of chemical induction, the midguts and fat bodies tissues were taken from all treatments including control and stored at −80 °C for RNA extraction. Each treatment had three biological replicates.

### 4.10. RNA Extraction and cDNA Synthesis

The fourth instar larvae of *S. exigua* were dissected to harvest midguts, fat bodies, and cuticle. Total RNA was prepared using the Trizol reagent according to the manufacturer’s protocol (Takara, Japan), and treated with DNase I (Qiagen, Valencia, CA, USA). The concentration and purity of total RNA were determined by a NanoDrop^®^ spectrophotometer (Thermo Fisher, MA, USA) and RNA integrity was examined by agarose gel electrophoresis. First-strand complementary DNA (cDNA) was synthesized by using TransScript^®^ One-Step gDNA Removal and cDNA Synthesis SuperMix in 20 µL reactions containing 1 µg of total RNA (500 ng), 1 µL Anchord Oligo(dT)_18_ Prime (0.5 µg/µL), 10µL 2×TS Reaction mixture, TransScript^®^ RT/RI Enzyme Mix and gDNA Remover at 42 °C for 30 min. Three independent RNA preparations representing three biological replicates were used for cDNA synthesis.

### 4.11. Quantitative Real-Time PCR

The expression levels of cytochrome P450 genes were quantified by quantitative real-time PCR (qRT-PCR) using an MYiQ’ RT-PCR system Bio-Rad, California, USA) and Real Master Mix 2xSYBR Green qPCR mix (Aidlab Biotechnologies Co., Ltd., China). QRT-PCR of each cDNA sample and template-free was performed in triplicate. Specific primers of *CYP6AB14* and *CYP9A98* were designed for qPCR (Table 2). Reaction volume of 20 µl was used (0.5 µL of each primer 10µM, 1 µL cDNA, 8 ul ddH20 and 10 µL 2x cyber master mix for quantification using the following cycling parameters: 94 °C for 3 min, followed by 40 cycles of 94 °C for 15 sec, 57–60 °C for 30 s and 70 °C for 30 s. For each gene, a serial dilution from 10- to 1000-fold of each cDNA template was performed to assess the efficiency of PCR. The relative expression values were calculated using the 2^−ΔΔCT^ methods as previously described by Livak and Schmittgen (2001). Results were expressed as the mean expression ratio (±S.E.) and each sample, including control, was run in three replicates. One-way analysis of variance (ANOVA) and the Tukey HSD test for the significant difference was performed to determine the statistical difference between means (SPSS, version 19).

### 4.12. Preparation of dsRNA

For dsRNA synthesis, a 405 bp fragment of *CYP6AB14* (GenBank accession KX443423) and a 358 bp fragment of *CYP9A98* (GenBank accession KX443440) were amplified by PCR. The primers used for the *CYP6AB14*, *CYP9A98*, and *dsRED* amplifications were designed to add the T7 polymerase promoter sequence to the 5′ end of each strand (Table 2). Similarly, the *dsRED* as a control was prepared using the same method by designing the two pairs of primer (T7RED-F and RED-R, RED-F and T7RED-R) (Table 2). The *dsRED* reference template was provided by the Dr. Xianchun Li laboratory (University of Arizona). The *dsCYP6AB14*, *dsCYP9A98*, and *dsRED* were prepared from the purified PCR-generated templates according to the instructions and method provided with the T7 RiboMax Express RNAi System kit (Promega, Madison, WI, USA). The dsRNA was then purified by using MEGA clear^TM^ Kit (Ambion). The resulting dsRNAs from all genes including control gene were quantified by a NanoDrop^®^ spectrophotometer (Thermo Fisher, MA, USA) and integrity was analyzed by agarose gel electrophoresis, and then stored at −80°C prior to use.

### 4.13. Administration of dsRNA by Droplet-Feeding

To prevent the damage to *S. exigua* larvae, we used a droplet-feeding method for RNAi interference [24,53,54]. For RNAi bioassays, double-stranded RNAs (dsRNA) dissolved in diethylpyrocarbonate (DEPC)-treated water. The fourth instar larvae were placed individually in 12-orifice tissue culture plates and starved for 6 h. The dsRNA solution (500 µg/µl) was configured by dissolving in DEPC treated water. The starved larvae were placed individually in 12-orifice tissue culture plate containing the artificial diet and one drop 0.5 µl (500 µg/µl) of dsRNA solution was placed near each larval mouth using a Microliter Syringe Beijing Karaltay Scientific Instruments Co., Ltd. Twenty-four hours after feeding on dsRNA larvae were subjected to toxicity analysis.

For toxicity analysis, after 24 h of dsRNA post-feeding, 60 *S. exigua* larvae for each independent treatment (Each of three replicate consisted of 20 larvae) were transferred individually into 12-orifice tissue culture plate containing artificial diets supplemented with 0.1 % gossypol, LC_50_ concentration of deltamethrin 0.887mg/L for 72 h, and 0.1 % gossypol for 24 h followed by deltamethrin for 72 h and standard diet. A non-supplemented diet was used as a control group. The mortality data were recorded at 48 and 72 h after feeding of dsRNA on different treatments including control. All experiments were performed in triplicate.

### 4.14. Combined Effects of dsRNA on Mortality

Similarly, the combined effect of two target genes (*CYP6AB14* and *CYP9A98*) was analyzed by mixing an equal volume of each dsRNA to obtain a 1.0 µL (1000 µg/µL) concentration. The feeding assay was performed for all treatments by a droplet-feeding method as described above.

For toxicity analysis, after 24 h of dsRNA feeding, 60 *S. exigua* larvae for each independent treatment (Each of three replicate consisted of 20 larvae) were transferred individually into 12-orifice tissue culture plate containing artificial diets supplemented with 0.1 % gossypol, LC_50_ concentration of deltamethrin 0.887mg/L for 72 h, and 0.1 % gossypol for 24 h followed by deltamethrin for 72 h and standard diet. A non-supplemented diet was used as a control group. The mortality data were recorded at 48 and 72 h after feeding of dsRNA on different treatments including control. All experiments were performed in triplicate.

### 4.15. Analysis of the Silencing Effect

To assess the expression level associated with each treatment by using qRT-PCR, approximately fifteen midguts and fat bodies (for each of three replicate) from surviving larvae were collected for total RNA extraction at two times (48 and 72 h). Quantitative RT–PCR was performed as described earlier.

### 4.16. Statistical Analysis

All data were analyzed using the SPSS 20.0 Software Package (SPSS Inc., Chicago, IL, USA). One-way ANOVA followed by the Tukey HSD test was employed to analyze differences between tissues and developmental stages. A Student’s t-test was used to analyze data from the RNAi and feeding experiments with chemicals.

## Figures and Tables

**Table 1:** The influences of gossypol ingestion and synergism effect of piperonyl butoxide (PBO) on the deltamethrin toxicity to *S. exigua* larvae.

Treatment	N	LC_50_ (mg a.i./L)	95% CL	Slope ± SE	df	χ2	SR
Control	420	0.887	0.75 ± 1.03	1.56 ± 0.14	4	1.66	1.7
Control + PBO	420	0.681	0.58 ± 0.79	1.61 ± 0.14	4	0.79	----
Gossypol	420	1.704	1.46 ± 1.98	1.71 ± 0.15	4	1.24	----
Gossypol + PBO	420	0.735	0.61 ± 0.87	1.47 ± 0.14	4	1.979	2.3

N: number of insects, CL: confidence limits, df: degrees of freedom, χ2: Chi-square value.

**Table 2:** Primers used in this study.

Function	Primer Name	Primer Sequence (5′-3′)
Real-Time PCR		
*CYP6AB14*	CYP6AB14-F	TCTTGATGCTGACTCGCTCA
	CYP6AB14-R	TACAGGCTTCCGGGAACATT
*CYP9A98*	CYP9A98-F	CTACCAGCATCTGCGTCAC
	CYP9A98-R	TTAGCCTACACCTTAACCAAT
*β-actin*	β-actin-F	ATCCTCCGTCTGGACTTGG
	β-actin-R	GCACGATTTCCCTCTCA
dsRNA synthesis		
*CYP6AB14*	T7CYP6AB14-F1	ggatcctaatacgactcactataggATGGGCTTTTCCAATCTTTC
	CYP6AB14-R1	GCTTAAACGTGCACAAGACAG
	CYP6AB14-F2	ATGGGCTTTTCCAATCTTTC
	T7CYP6AB14-R2	GCTTAAACGTGCACAAGACAGggatcctaatacgactcactatagg
*CYP9A98*	T7CYP9A98-F1	ggatcctaatacgactcactataggGAGAACTTCCTCAACCATCCTAA
	CYP9A98-R1	TGATTCCGCTAAGTATCTTTCCC
	CYP9A98-F2	GAGAACTTCCTCAACCATCCTAA
	T7CYP9A98-R2	TGATTCCGCTAAGTATCTTTCCCggatcctaatacgactcactatagg
*dsRED*	T7dsRED-F1	ggatcctaatacgactcactataggGCAAGCTATGCATCCAACGCGTTGGG
	dsRED-R1	CAAGCTATGCATCCAACGCGTTGGGAG
	dsRED-F2	GCAAGCTATGCATCCAACGCGTTGGG
	T7dsRED-R2	CAAGCTATGCATCCAACGCGTTGGGAGggatcctaatacgactcactatagg

## Supplementary Materials

Supplementary materials can be found at https://www.mdpi.com/1422-0067/20/9/2248/s1.
