# Supplementary material for: Knock-Down of Gossypol-Inducing Cytochrome P450 Genes Reduced Deltamethrin Sensitivity in Spodoptera exigua (Hübner)
Source: Int J Mol Sci. 2019 May 7;20(9):2248. doi: 10.3390/ijms20092248 (PMC6539524; doi:10.3390/ijms20092248)
Supplement: Supplementary file 1 [file ijms-20-02248-s001.pdf]

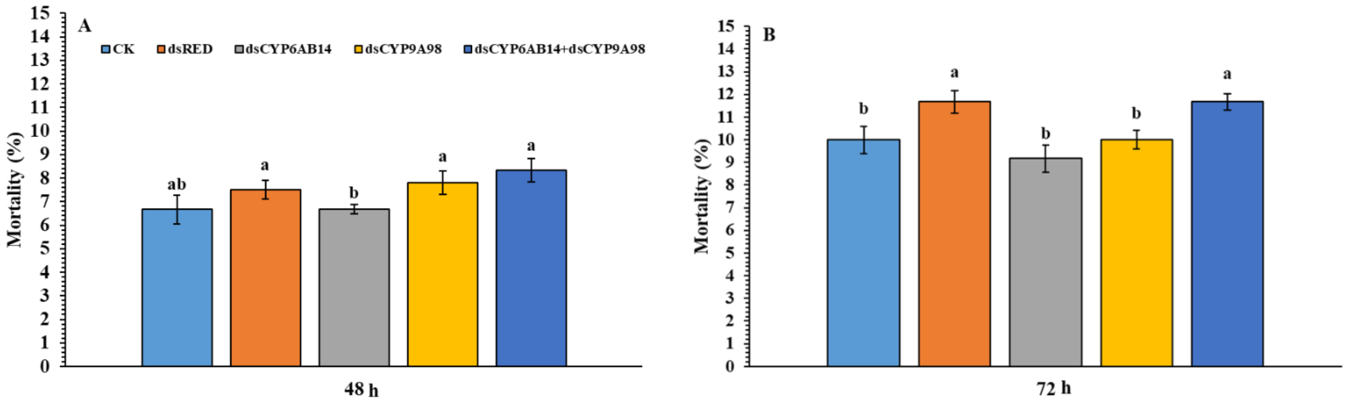

Figure S1. Effects of dsCYPAB14 and dsCYPA98 feeding on the susceptibly of fourth instar *S. exigua* larvae. Following the droplet-feeding with dsCYPAB14+dsCYPA98 or dsRED for 24 h the exposed larvae were transferred individually into 12-oriface tissue culture plate containing artificial diets without chemicals for 48 (A) and 72 h (B). Data shown are means ± SE derived from three biological replicates. Different letters above bars indicate significant differences ($p < 0.05$) according to the Tukey HSD test.
